# A SARS–CoV-2 Spike Receptor Binding Motif Peptide Induces Anti-Spike Antibodies in Mice andIs Recognized by COVID-19 Patients

**DOI:** 10.3389/fimmu.2022.879946

**Published:** 2022-05-26

**Authors:** Federico Pratesi, Fosca Errante, Lorenzo Pacini, Irina Charlot Peña-Moreno, Sebastian Quiceno, Alfonso Carotenuto, Saidou Balam, Drissa Konaté, Mahamadou M. Diakité, Myriam Arévalo-Herrera, Andrey V. Kajava, Paolo Rovero, Giampietro Corradin, Paola Migliorini, Anna M. Papini, Sócrates Herrera

**Affiliations:** ^1^ Department of Clinical and Experimental Medicine, University Hospital of Pisa, Pisa, Italy; ^2^ Interdepartmental Laboratory of Peptide and Protein Chemistry and Biology, Department of NeuroFarBa, University of Florence, Sesto Fiorentino, Italy; ^3^ Interdepartmental Laboratory of Peptide and Protein Chemistry and Biology, Department of Chemistry “Ugo Schiff”, University of Florence, Sesto Fiorentino, Italy; ^4^ Department of Immunology, Caucaseco Scientific Research Center, Cali, Colombia; ^5^ Department of Pharmacy, University of Naples Federico II, Naples, Italy; ^6^ Immunogenetic Laboratory and Parasitology, University of Sciences, Techniques and Technologies of Bamako (USTTB), Bamako, Mali; ^7^ Department of Nephrology, University Hospital Regensburg, Regensburg, Germany; ^8^ Department of Immunology, Malaria Vaccine and Drug Development Center, Cali, Colombia; ^9^ CRBM, University of Montpellier, CNRS, Montpellier, France; ^10^ Biochemistry Department, University of Lausanne, Lausanne, Switzerland

**Keywords:** SARS-CoV-2, receptor binding motif, COVID-19, immunized animals, neutralizing Abs, spike (S) protein

## Abstract

The currently devastating pandemic of severe acute respiratory syndrome known as coronavirus disease 2019 or COVID-19 is caused by the coronavirus SARS-CoV-2. Both the virus and the disease have been extensively studied worldwide. A trimeric spike (S) protein expressed on the virus outer bilayer leaflet has been identified as a ligand that allows the virus to penetrate human host cells and cause infection. Its receptor-binding domain (RBD) interacts with the angiotensin-converting enzyme 2 (ACE2), the host-cell viral receptor, and is, therefore, the subject of intense research for the development of virus control means, particularly vaccines. In this work, we search for smaller fragments of the S protein able to elicit virus-neutralizing antibodies, suitable for production by peptide synthesis technology. Based on the analysis of available data, we selected a 72 aa long receptor binding motif (RBM_436-507_) of RBD. We used ELISA to study the antibody response to each of the three antigens (S protein, its RBD domain and the RBM_436-507_ synthetic peptide) in humans exposed to the infection and in immunized mice. The seroreactivity analysis showed that anti-RBM antibodies are produced in COVID-19 patients and immunized mice and may exert neutralizing function, although with a frequency lower than anti-S and -RBD. These results provide a basis for further studies towards the development of vaccines or treatments focused on specific regions of the S virus protein, which can benefit from the absence of folding problems, conformational constraints and other advantages of the peptide synthesis production.

## Introduction

The current SARS-CoV-2 (severe acute respiratory syndrome coronavirus 2) pandemic has resulted in devastating social and economic consequences worldwide, in addition to an enormous public health burden. Coronaviruses are single-stranded RNA-enveloped viruses (1). Although this type of viruses is frequently associated with a common cold with mild symptoms in humans, some of them can cause severe respiratory infection and death, mainly in elderly patients and in individuals with several comorbidities, primarily diabetes, obesity, hypertension and other cardiovascular disorders (2–4).

The ongoing coronavirus disease 2019 (COVID-19) is considered one of the world's worst pandemics, with more than 400 million cases and 5.8 million human deaths reported as of February 2022 (5). Since the beginning of the COVID-19 pandemic, the scientific community has focused intense efforts on studying the virus biology, the disease manifestations and management and its prevention (6, 7). In a short time, the SARS-CoV-2 genome, the specificity of its overall structural organization and the atomic 3D structure of the most significant proteins were revealed (8, 9).

One of the critical proteins is a trimeric spike (S) protein that allows this virus to penetrate host cells and cause infection. The S protein trimers protrude from the outer bilayer leaflet and form a characteristic crown-like halo surrounding the viral particle (hence, "corona"). The importance of the SARS-CoV2 S-protein is that it is a large self-assembled homo-trimer protein of about 1,250 aa (8, 9), expressed on the virus membrane and responsible for the virus-cell invasion. The protein is composed of two functional subunits, S1 and S2. The S1 subunit, which forms the globular head of the S protein trimer, contains the receptor-binding domain (RBD) that specifically interacts with the host receptor angiotensin-converting enzyme 2 (ACE2).

The S2 subunits form the stalk of the trimer embedded into the viral envelope. When the S protein binds to the ACE2 receptor, proteases located on the host cell membrane trigger the dissociation of S1 fragments and induce an irreversible refolding of the S2 trimer. The structural rearrangement of S2 brings together the viral and cellular membranes, leading to the fusion of the two bilayers. The atomic 3D structure of the S trimer in the prefusion conformation, the S2 trimer in the post-fusion conformation, and the RBD-ACE2 complex have been determined (10–12) and all have contributed to developing means to control virus spreading. Specifically, these features of the S protein led vaccine companies to choose it for vaccine development (13, 14).

The RBD is a monomeric domain of a smaller size (220 aa) that folds in the same stable 3D structure as part of the complete S protein and as a separate domain (15). Antiviral antibodies and cell mediated responses of multiple specificities are produced during SARS-CoV-2 infection and appear to contribute to protection (16). RBD is not only essential for virus invasion of host cells, but also targets neutralizing antibodies generated during SARS-CoV-2 infection; therefore, RBD represents another promising vaccine candidate (8, 17, 18).

While the rate of infections and deaths rapidly increased worldwide, significant efforts were invested in developing effective tools to promptly confirm diagnosis of the infection i.e., highly sensitive and specific molecular diagnostic methods (19). Likewise, given that vaccines are the primary medical option and most cost-effective means for global control of the pandemic, an unprecedented effort to develop anti-COVID-19 vaccines led to the production, clinical evaluation and approval by regulatory agencies of multiple vaccines. Along this line, given the critical functions of the S protein, the viral surface location, and the availability of detailed structural information, this protein was chosen for vaccine development (9, 20, 22).

As of February 2022, more than ten billion vaccine doses had been delivered globally, and ~60% of the world population had received at least one vaccine dose (5). Moreover, despite specific antiviral drugs having been elusive until recently, two novel antiviral medicines have already been approved by the United States Food and Drug Administration (FDA). Molnupiravir produced by Merck (23), and Nirmatrelvir/Ritonavir (Paxlovid) produced by Pfizer (24) are medicines for oral administration, with high effectiveness to reduce disease severity and prevent deaths (25).

Although the most extensively used vaccines have shown high protective efficacy, their effectivity, particularly the antibody response's longevity and the virus-neutralizing function, appears short-lasting, suggesting the need for new vaccine formulations. Based on the recent advances in understanding the structure and function of S protein, and with the aim of identifying highly effective virus proteins/fragments this work concentrate on further characterization of the S protein, focusing on shorter fragments/domains with vaccine potential. We selected the S-ACE2 receptor binding motif (RBM_436-507_) which was produced as a single synthetic peptide, along with shorter sequences which were compared in their antigenicity and immunogenicity using sera from humans naturally exposed to COVID-19, and sera from immunized animals. Selected sera were also analyzed for their neutralization activity.

## Materials and Methods

### Recombinant S and RBD Proteins Production 

Since the S trimer is described as the primary protein responsible for inducing a protective immune response against the SARS-CoV-2 virus, first we produced a secreted and soluble form of this protein self-assembled in the trimer using Chinese Hamster Ovary (CHO) cells as previously described (26). Briefly, the transmembrane domain and the C terminal intracellular tail were removed and replaced by a T4 foldon DNA sequence and an 8xHis tag. A signal peptide sequence was added. To stabilize the prefusion structure of the S trimer in our constructs, we deactivated the original RRA furin cleavage site R by changing it to RGSA. We introduced amino-acid mutations K986P/V987P ("2P") as suggested elsewhere (12). The construct used in this work had the D614G mutation shared by most of the SARS-CoV-2 variant of concern (B.1.1.7 - Alpha, B.1.351 - Beta, B.1.617.2 - Delta, B.1.1.529 - Omicron) widely spread during the 2020-2021 pandemic (27). This S protein construct was established to form trimers predominantly folded in the prefusion conformation (26). In addition, the RBD of the S protein (aa 319-541) was produced as a recombinant product (26) and a series of peptides covering the BIP sequence were synthesized and analyzed.

### Peptide Synthesis, Purification and Characterization 

Peptide sequences corresponding to the full RBM_436-507_ length (72 aa) as well as shorter fragments of 20-22 amino acids (P11-P16) described in **Table 1** were synthesized and analyzed. Single cysteine residues in peptides P11, P12, and P13 (486-507, 476-495 and 466-485 of S protein, respectively) were replaced with serine to avoid unwanted spontaneous formation of disulfide dimers. Peptides were prepared by microwave-assisted solid-phase peptide synthesis (MW-SPPS), cleaved from the resin and, in the case of RBM_436-507_ and P12, oxidized in solution with H_2_O_2_ at pH 9.0. (28) Purifications were performed by flash chromatography followed by semi-preparative HPLC to achieve purity >70% (RBM_436-507_ and P16) or >87% (P11-P15). Final products were characterized by analytical UHPLC coupled with ESI single quadrupole mass spectrometry and/or MALDI-ToF analysis. Analytical data and details on the synthesis and purification procedures are available as **Supplementary Information**.

**Table 1:** Synthesized RBM peptide sequences.

Name	Sequence	Amino acids
**RBM_436-507_**	Ac-WNSNNLDSKVGGNYNYLYRLRKSNLKPFERDISTEIYQAGSTPCNGVEGFNCYFPLQSYGFQPTNGVGYQP-NH_2_	436-507
**P11**	Ac-FNSYFPLQSYGFQPTNGVGYQP-NH_2_	486-507
**P12**	Ac-GSTPCNGVEGFNCYFPLQSY-NH_2_	476-495
**P13**	Ac-RDISTEIYQAGSTPSNGVEG-NH_2_	466-485
**P14**	Ac-FRKSNLKPFERDISTEIYQA-NH_2_	456-475
**P15**	Ac-GGNYNYLYRLFRKSNLKPFE-NH_2_	446-465
**P16**	Ac-WNSNNLDSKVGGNYNYLYRL-NH_2_	436-455

Underlined sequences in peptides P3 and P12 represent disulfide bridges. Serine residues (S) highlighted in red in peptides P11 and P13 replace native Cysteines.

### Conformational Studies by Circular Dichroism

The CD spectrum of the RBM_436-507_ peptide was recorded using quartz cells of 0.1 cm path length with a JASCO J-710 CD spectropolarimeter at 25 °C. The spectrum was measured in the 260−190 nm spectral range, 1 nm bandwidth, 64 accumulations, and 100 nm/min scanning speed. The peptide was dissolved in water to a concentration of 12 μM. The secondary structure content of the peptide was predicted using the online server for protein secondary structure analyses DichroWeb (29). Input and output units and the wavelength step were θ (mdeg) and 1.0 nm, respectively.

The mean residue molar ellipticity [Θ]MR (Y-axis label) was calculated, which is defined as:


[Θ]MR=Θ/(10 x Cr x l)

where: Θ is ellipticity in mdeg, Cr is the mean residue molar concentration, l is the cell path in cm, and Cr = (n x 1000 x Cg)/Mr

where: n is the number of peptide bonds (residue), Cg is the macromolecule concentration (g/ml), Mr is the molecular weight of the peptide.The algorithm used was CDSSTR, and the reference database was set-7 (30).

The normalized root means square deviation (NRMSD) was 0.035.

### Human Blood Samples 

A clinical protocol was developed, submitted to and approved by the local Ethical Committees (CEAVNO, Approval # 17522) in Italy and (CECIV, approval # 04-2020) in Colombia. Whole blood (10 mL) was collected from COVID-19 patients from both Italy and Colombia. Samples were collected by arm venipuncture using dry tubes after hospitalization, and upon the patient's written informed consent, socio-demographic data and clinical manifestations were recorded. SARS-CoV-2 infection was confirmed by RT-PCR. Blood was fractionated, and sera were collected and kept frozen at -20^o^C until use for serology.

### Mice Immunization and Sera Collection 

A total of 30 male and female, 6-8 weeks old BALB/c mice of 20 ± 5 g of body weight were randomly selected and distributed in three groups (A, B and C) of 10 animals each. Each group was further divided into experimental (Exp) and control (Ctrl) sub-groups of five mice each and were further immunized with SARS-CoV-19 S (group A) or RDB (group B) recombinant proteins as well as with the synthetic RBM_436-507_ peptide (group C). Each group of mice was immunized subcutaneously (s.c.) at the base of the tail on days 0, 20 and 40 with 20μg of each antigen diluted in 50 μL PBS and emulsified in Montanide ISA-51 (Seppic Inc., Paris, France) according to the manufacturer's recommendations. Mice were bled from submandibular veins on days 1-2 before the first and third immunizations, 20 days after the third dose and every 60 days until day 140. Whole blood (~100 μL) was collected, and sera were separated by centrifugation and stored frozen at -20°C until use for serological analyses. Animal studies were carried out at the Caucaseco Research Center in Cali (Colombia) and approved by the Animal Ethics Committee of MVDC in Colombia. Animal care, housing, and handling were performed according to institutional guidelines and following the National Institutes of Health Guide for the Care and Use of Laboratory Animals.

### Serological Analyses

#### Reactivity of Mouse Antibodies to S and RBD Proteins and RBM_436-507_

The reactivity of sera from mice immunized with the S, RBD and RBM_436-507_ was determined by ELISA, using as antigens the specific immunogens. Briefly, 96-well plates (Nunc-Immuno Plate, Maxisorp, Roskilde, Denmark) were coated with one µg/mL RBM_436-507_, RBD and Spike Trimer protein, pH 7.4 at 4°C, overnight. After plates were blocked with 5% skim milk solution [PBS 1X, 0.05% Tween 20, (PBS-T)], serum samples were added at 1:100 or three-fold serial dilutions starting at 1:100 in 2.5% skim milk in PBS-T and were incubated for 1 hour. Plates were then washed and incubated with alkaline phosphatase-conjugated anti-mouse IgG antibody (Sigma Chemical Co., St Louis, MO) at a 1:1000 dilution for 1 hour. Reactions were revealed with para-nitrophenyl phosphate substrate (*p*-NPP) (Sigma Aldrich) and read at 405 nm wavelength (Dynex Technologies, Inc., MRX Chantilly, VA).

### ELISA Assays to Analyze Anti-Spike, Anti-RBD and Anti-RBM_436-507_ Human Antibodies

Nunc Maxisorp polystyrene plates were coated with Spike Trimer (Excellgene, Monthey, Switzerland) or RBD (Excellgene, Monthey, Switzerland) at 1 μg/ml in PBS pH 7.4 (50 μl/well) overnight at 4°C; peptide RBM_436-507_ coating was at 2 μg/ml in Carbonate buffer, pH 9.6; 20-mers P11-P16 at 10 μg/ml in PBS, pH 7.4. After blocking for 1 hr at room temperature (RT) with PBS pH 7.4, BSA 3% (A4503 - Merck KGaA, Darmstadt, Germany), sera diluted 1/100 in PBS pH 7.4, BSA 1%, Tween-20 0.05% were incubated on the plate (50 μl/well) for 2 hours at RT. After 3 washings with PBS Tween-20 0.05% (150 μl/well), goat anti-human IgG HRP (A0293 - Merck) diluted 1:5000 in PBS BSA 1% Tween-20 0.05% was added to the plates at 50 μl/well and incubated for 2 hours. For IgM and IgA determination, goat anti-human IgM HRP conjugate (A0420 – Merck) or goat anti-human IgA HRP conjugate (A0295 - Merck) diluted 1:20,000 in PBS, BSA 1%, Tween 0.05% were added to the plates. After three washings with PBS Tween-20, 0.05%, enzymatic activity was measured at 450 nm after TMB addition (T4444 - Merck) and blocked by H_2_SO_4_ 1M.

### Inhibition of ACE Binding to RBD With Anti-RBM_436-507_ Specific Human Antibodies

The ability of anti-RBM_436-507_ antibodies to inhibit the binding of ACE2 to RBD was evaluated using a modification of the SPIA commercial kit (Diametra Srl, Spello, Pg - Italy, ImmunoDiagnostic System Group). Anti-RBD antibodies were used as a positive control. Anti-N1 (20-mer linear peptide of SARS-CoV-2 nucleocapsid, aa 366-388) and anti-TT (tetanus toxoid) antibodies were used as virus-related and -unrelated negative controls. Specific antibodies were eluted from four sera with high anti-COVID-19 antibody titers using polystyrene plates coated with RBD, RBM_436-507_, N1 and TT. Briefly, the plates were blocked with PBS BSA 3%, and COVID-19 sera diluted 1/50 in PBS BSA 1% Tween-20 0.05% and incubated for 2 hours at RT. Plates were washed three times with PBS Tween-20 0.05%, and bound antibodies were eluted with 200μl PBS pH 3.0 and immediately neutralized at pH 7.4 with basic phosphate buffer. The concentration of eluted antibodies was evaluated by A_280_ absorbance measurement with Nanodrop, and binding to the respective antigen was confirmed by indirect ELISA. For ACE inhibition assay, anti-RBD, anti- RBM_436-507_, anti-N1 and anti-TT eluted antibodies were incubated onto Diametra SPIA plates coated with recombinant RBD. Calibrator and controls were loaded as per the manufacturer's instructions. Ready-to-use ACE2 conjugated with horseradish peroxidase was then added to the wells, and plates were incubated for 90 minutes at 37°C. After washings, plates were incubated with TMB for 15 minutes and acid stop solution was added before reading the absorbance at 450 nm. Results were expressed as percentage inhibition according to the manufacturer's instruction.

### Statistical Analysis 

Antibody titers were compared between mouse groups. A descriptive analysis was performed to evaluate differences in humoral immune responses within each group of mice. Kruskal-Wallis was performed to compare the antibody response to each protein, followed by Dunn's multiple comparison test. Results of anti-S, anti-RBD and anti- RBM_436-507_ antibodies were expressed as Odd Ratio (OR) of a positive internal control set at 1.0. A *p*-value < 0.05 was considered statistically significant. Data were analyzed and plotted using GraphPad Prism software (version 5.01; GraphPad Software Inc, San Diego, California, USA).

## Results

### Selection and Circular Dichroism Analysis of RBM_436-507_ Peptide 

**Figure 1:**
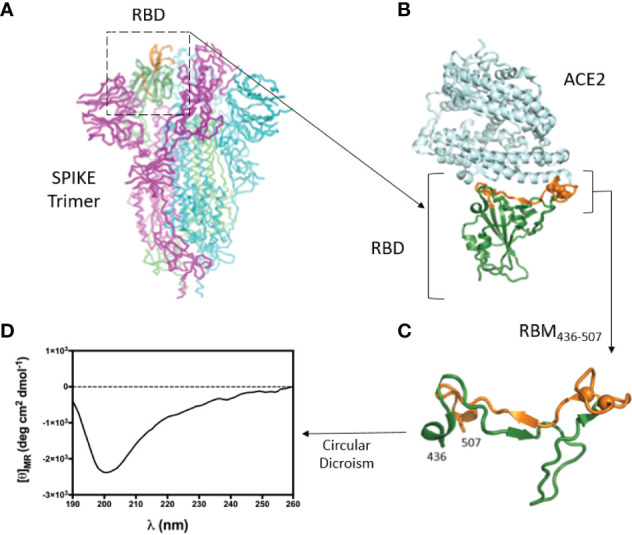
3D structure of the RBD-ACE2 complex and CD of RBM_436-507_. **(A)** Atomic 3D structure of the S trimer in the prefusion conformation (27). RBD is shown in ribbon representation (dark green). Region 476-507 is in orange. **(B)** Complex between RBD (green-orange) and ACE2 receptor (light cyan) (12). **(C)** Conformation of the RBM peptide (436-507) within the RBD. Cysteine residues are shown as spheres. **(D)** Circular Dichroism of RBM_436-507_ synthetic peptide used in this work.

To study the interaction between S and ACE2, we focused on the surface of the RBD involved in the ACE2 receptor binding, which should represent the target of the neutralizing antibodies. Our analysis of the 3D structure of the RBD-ACE2 complex showed that the large part of the RBD interacting surface, the Receptor Binding Motif (RBM), is composed of a 436-507 aa segment (**Figures 1A–C**). Since peptide synthesis technology has several advantages compared to recombinant proteins (31–34), we selected this RBM region for peptide synthesis and subsequent experimental studies. The central part of RBM_436-507_ should mimic well the native-like conformation due to a disulfide bond. The peptide flanking parts should be unstructured and highly flexible both in peptides as well as within the 3D structure of the S-protein. In addition to the critical surface localization of the RBM_436-507_ in the S protein, its amino acid sequence is specific to the SARS-CoV-2 and contains several predicted T-cell epitopes (33). The sequence of RBM_436-507_ (**Table 1**) was N-terminal acetylated and C-terminal amidated to avoid including terminal charged groups not present in the native protein.

The conformation of RBM_436-507_ in water at pH 7 was explored by CD spectrometry (**Figure 1D**). We then evaluated the antigenic properties of this peptide. The absence of a defined minimum around 200 nm, diagnostic of random coil conformation, is compatible with a certain degree of structuration of the peptide. The secondary structure content was predicted based on the CD spectrum using the online server for protein secondary structure analyses, DichroWeb (29): 2% helix, 30% β-strand, 19% β-turn, and 49% random coil. The relatively high percentage of β-strand conformation suggests the intriguing hypothesis that RBM_436-507_ peptide can partially preserve the extended conformation displayed along most of its sequence within the folded Spike protein (pdb code 6VXX) (8, 21).

### Immunogenicity of S, RBD and RBM_436-507_ in Mice 

**Figure 2:**
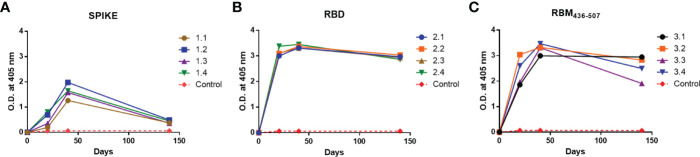
Immunogenicity of S, RBD and RBM_436-507_ in mice. Analysis of the antibody response in mice immunized at days 0, 20, 40. **(A)** Anti-S, **(B)** Anti-RBD and **(C)** Anti-RBM_436-507_. SD is < 20% of the mean.

As shown in **Figure 2**, sera from all immunized animals tested by ELISA at 1:100 dilution, in response to the S, RBD and RBM_436-507_ antigens, indicated specific IgG seroconversion after the first immunization dose. Furthermore, most of them displayed a boosting response after the second immunization dose, with the highest levels against the three proteins observed on day 40. However, while animals immunized with RBM_436-507_ and RBD developed similar high level antibody profiles (3.0 to 3.5 OD), mice immunized with the S protein displayed significantly lower responses (1.0 to 2.0 OD). For RBM_436-507_ and RBD, antibodies remained at high levels (>2.0 OD) after day 140, whereas antibodies against the S protein notably decreased (< 0.5 0D) during the same period. None of the control mice immunized with adjuvant alone seroconverted. The antibody titration (three-fold dilutions) using sera collected on day 140 indicated titers of 1:24,300, 1:72,900 to RBM_436-507_ and RBD respectively, and 1:900 to S (**Supplementary Material**, **Figure S1**).

### Reactivity of Mouse Antibodies to S, RBD and RBM_436-507_

**Figure 3:**
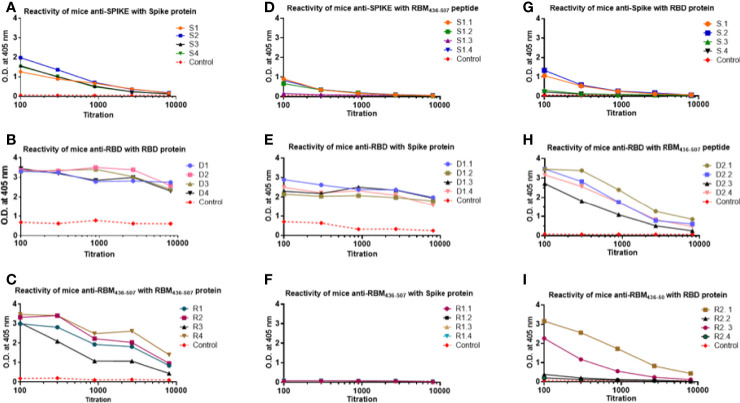
Homologous and cross reactivity of the S, RBD and RBM_436-507_ antigens with antibodies elicited upon mice immunization. **(A–C)** show reactivity of anti-S, anti-RBD and anti-RBM_436-507_ produced in mice with their homologous antigens. **(D, G)** show cross reactivity of anti-S with RBM and RBD antigens. **(E, H)** of anti-RBD with S and RBM antigens. **(F, I)**, of anti RBM436-507 with S and RBD, respectively. SD is < 20% of the mean.

The analysis of the homologous and cross recognition of the S, RBD and RBM_436-507_ antigens by antibodies elicited upon mice immunization is shown in **Figure 3**. ELISA results showed a high homologous sera reactivity but different reactivity with the other proteins/domains. Reactivity of sera diluted at 1:100 showed OD values ranging from 1.2 to 2.0 against the full-length S antigen, 3.2-3.5 to the RBD and 3.0-3-5 to the RBM_436-507_ fragment. The titration of this homologous reactivity indicated that final reactivity (OD 0,2) at 1:10^4^ dilution to the S protein (**Figure 3A**), whereas at the final dilution tested (1:10^4^) the OD values were higher for RBD (OD= 2.5-3.0) and RBM (0.5-1.7) (**Figures 3B, C**, respectively).

Regarding the analysis of the cross reactivity, anti-S antibodies displayed similar recognition of RBD and RBM_436-507_ (**Figure 3**), and the anti-RBD antibodies high recognition of both the S- and -RBM_436-507_ proteins, although the S-protein was better recognized. In contrast, for the anti-RBM_436-507_ antibodies, only two mice presented cross reactivity with end point of 1:10^4^ whereas the remaining animals of the group presented only weak reactivity at 1:100 dilution. Notably, these antibodies did not cross react with the S-protein (**Figures 3D–I**).

**Figure 4:**
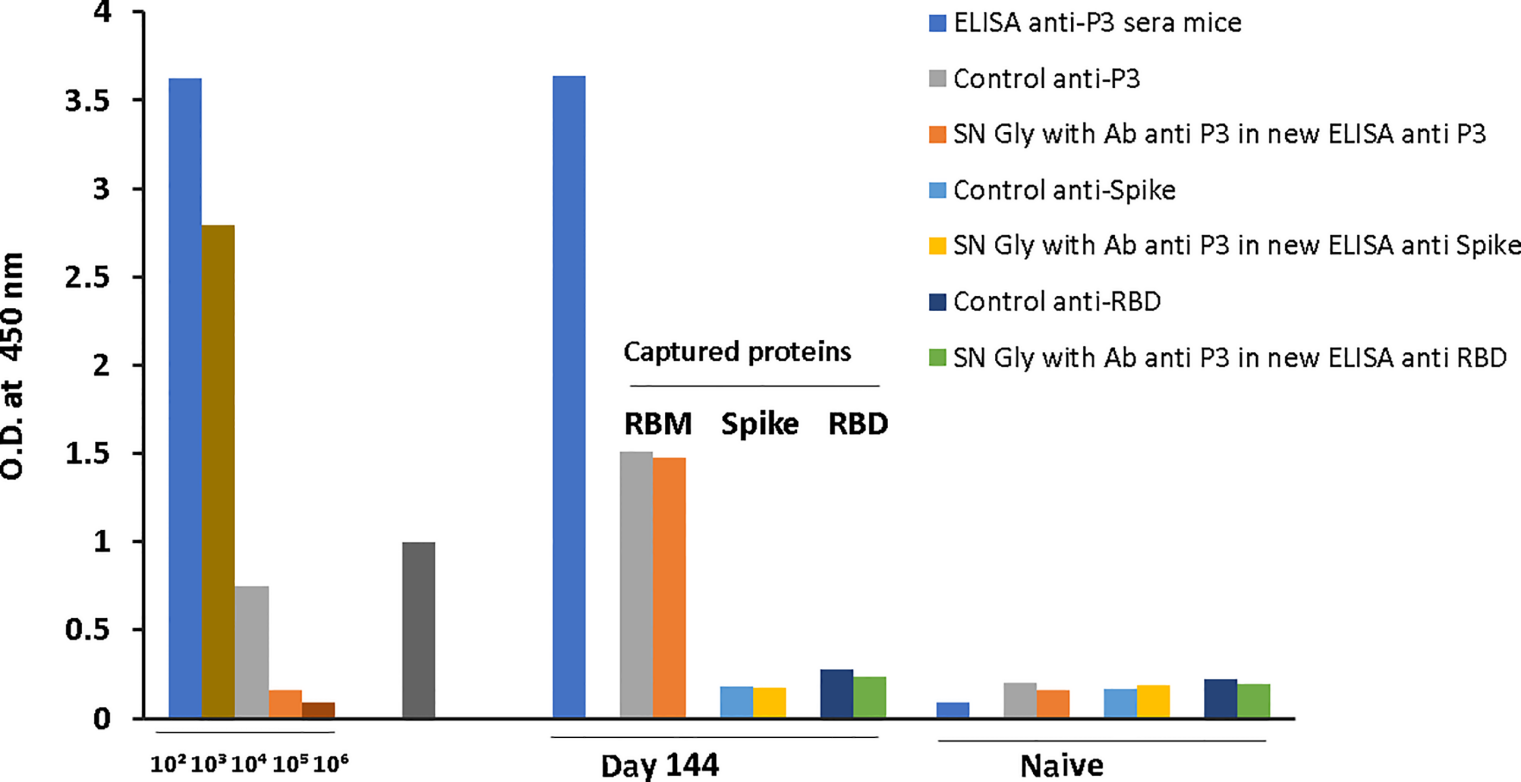
Cross-reactivity of S and RBD with ELISA captured RBM_436-507_. ELISA captured mice anti-RBM_436-507_ antibodies were eluted with Gly pH 2.5 and used to determine the reactivity with RBM (homologous), and with S and RBD (heterologous) antigens ELISA reaction was developed using rabbit anti-mouse alkaline phosphatase conjugate.

The final reactivity titer of the anti-S antibodies was 1:10^4^ against the S protein, and 1.8x10^3^ against RBD and RBM_436-507_. In the case of RBD, mouse immunization elicited a vigorous antibody response (**Figure 3**) with high optical densities even at 1:10^4^ dilution. Although reactivity to the S protein and the RBM_436-507_ peptide were lower, recognition remained significant even at dilutions of 1:10^4^ and 5:10^3^, respectively. Sera from mice immunized with RBM_436-507_ peptide also displayed high reactivity with the homologous peptide and the RBD protein; however, these sera did not react with the S protein (**Figure 3**). We further analyzed reactivity of anti-RBM_436-507_ antibodies upon solid-phase capture on ELISA plates followed by glycine elution with its homologous peptide, the RBD and the S proteins. As shown in **Figure 4**, while there was significant reactivity of eluted antibodies with RBM_436-507_, no recognition of the motif on the RBD and S proteins was observed.

### Evaluation of Anti- RBM_436-507_ Antibodies in Humans

**Figure 5:**
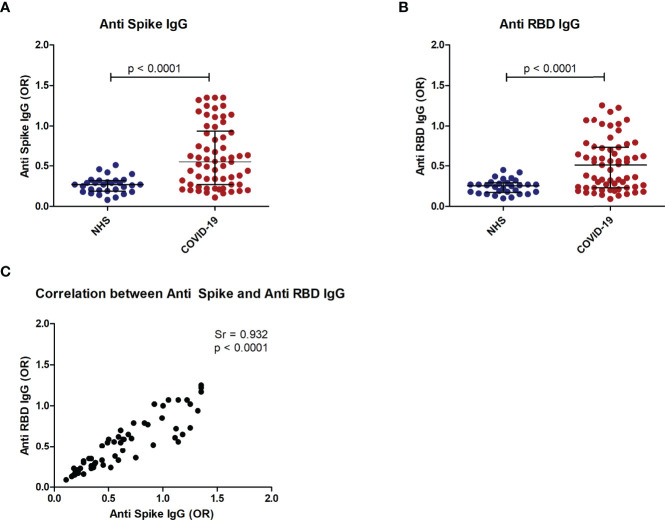
Anti-Spike and anti-RBD antibodies in COVID-19 patients. Distribution of anti-S IgG **(A)** and anti RBD IgG **(B)** in COVID-19 patients as compared to normal controls (NHS). Correlation of anti-S IgG and anti-RBD IgG in COVID-19 patients **(C)**. p < 0.05 was considered significant.

Patient sera were first screened by ELISA using S and RBD proteins and compared to a group of pre-pandemic normal sera. IgG antibody levels higher than the 97.5^th^ percentile of normal sera were detected in 45% (29/64) of patient sera on S and in 53% (34/64) on RBD (**Figures 5A, B**). A strong positive correlation (p<0,0001) was observed between antibody levels for the two recombinant proteins (**Figure 5C**).

**Figure 6:**
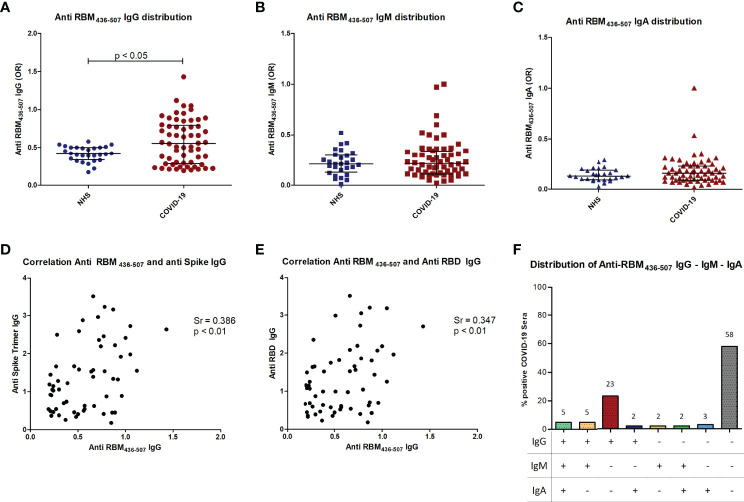
Anti-RBM_436-507_ Ig isotypes in COVID-19 patients. Distribution of anti RBM IgG **(A)**, IgM **(B)** and IgA **(C)** in COVID-19 patients is shown compared to normal controls (NHS). Correlation of anti-RBM IgG with anti-Spike **(D)** or anti-RBD **(E)** IgG in COVID-19 patients **(D)**. Distribution of anti-RBM antibody isotypes **(F)**. p < 0.05 was considered significant.

It has been shown that low pH affects spike structure, favoring a closed conformation of the trimer (34), affecting epitope exposure (16). We thus performed the ELISA assay at acidic pH, obtaining a similar level of antibodies in patient sera (**Supplementary Figures 2A, B**). Sera from COVID-19 patients and normal subjects were tested by ELISA using RBM immobilized on polystyrene plates (see *Materials and Methods* for details). IgG anti-RBM_436-507_ higher than the 97.5^th^ percentile of the healthy population was detected in 21/60 (35%) of the COVID-19 patients. IgG antibody levels were significantly higher in patients than in controls (p<0.05) (**Figure 6A**) and were correlated with anti-S and anti-RBD antibody levels (p < 0.01) (**Figures 6D, E**). Anti RBM_436-507_ of IgM and IgA isotype were also evaluated, with IgM anti- RBM_436-507_ detected in 7/60 (11.6%) and IgA in 6/60 (10%) (**Figures 6B, C**). IgM and IgA antibody levels were not significantly different in COVID-19 patients and controls. There was coexpression of anti-RBM_436-507_ Ig isotypes in COVID-19 samples (**Figure 6F**).

### Epitope Mapping and Functional Activity of Murine and Human Anti-RBM_436-507_ Antibodies 

**Figure 7:**
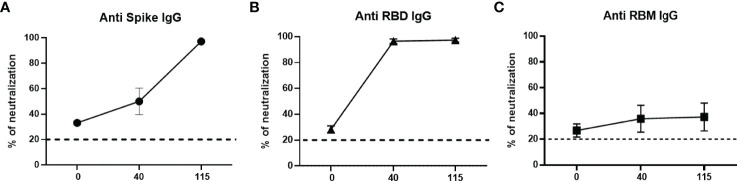
Neutralizing ability of antibodies in mice. Neutralizing ability of anti-S **(A)**, anti-RBD **(B)** and anti-RBM **(C)** antibodies from immunized mice. Results are shown as the percentage of inhibition of specific antibodies at different days (0, 40, and 115) post-immunization.

The analysis of the neutralizing activity of antibodies elicited by mouse immunization showed that, for mice immunized with S and RBD, the neutralization was significantly boosted after the second and third doses. Both S and RBD sera induced total neutralization after the third dose and remained high until the last test on day 115. In contrast, antibodies to RBM_436-507_ reached 40% neutralization, which remained at that level until day 115 (**Figures 7A–C**).

To determine whether RBM_436-507_ represents a target of neutralizing antibodies in natural conditions, we first carried out an extensive ELISA analysis of sera from both COVID-19 patients and immunized mice, and second, we compared the ACE2-RBD binding neutralization by antibodies to the whole RBD and to RBM_436-507_. In the ELISA analysis of human sera (n= 100) from COVID-19 patients 35 (35%) reacted with the RBM_436-507_ indicating a lower reactivity than the same sera with the S and RBD. Positive samples displayed distinct reactivity with different regions of RBM_436-507_, more frequently with the N-terminal portion (P15-P16). Neutralizing activity of anti-RBM_436-507_ antibodies has been evaluated by inhibition of RBD binding to ACE2, an assay considered a SARS-CoV-2 surrogate virus neutralization test (35–37). Neutralizing antibodies may bind to sequences exposed both in the closed and the open conformation of the S protein or only in the open one; most of these sequences are comprised in RBM_436-507_. In contrast to human patients, mice immunized with RBM_436-507_ presented good recognition of RBM_436-507_ and RBD but no reactivity with S.

**Figure 8:**
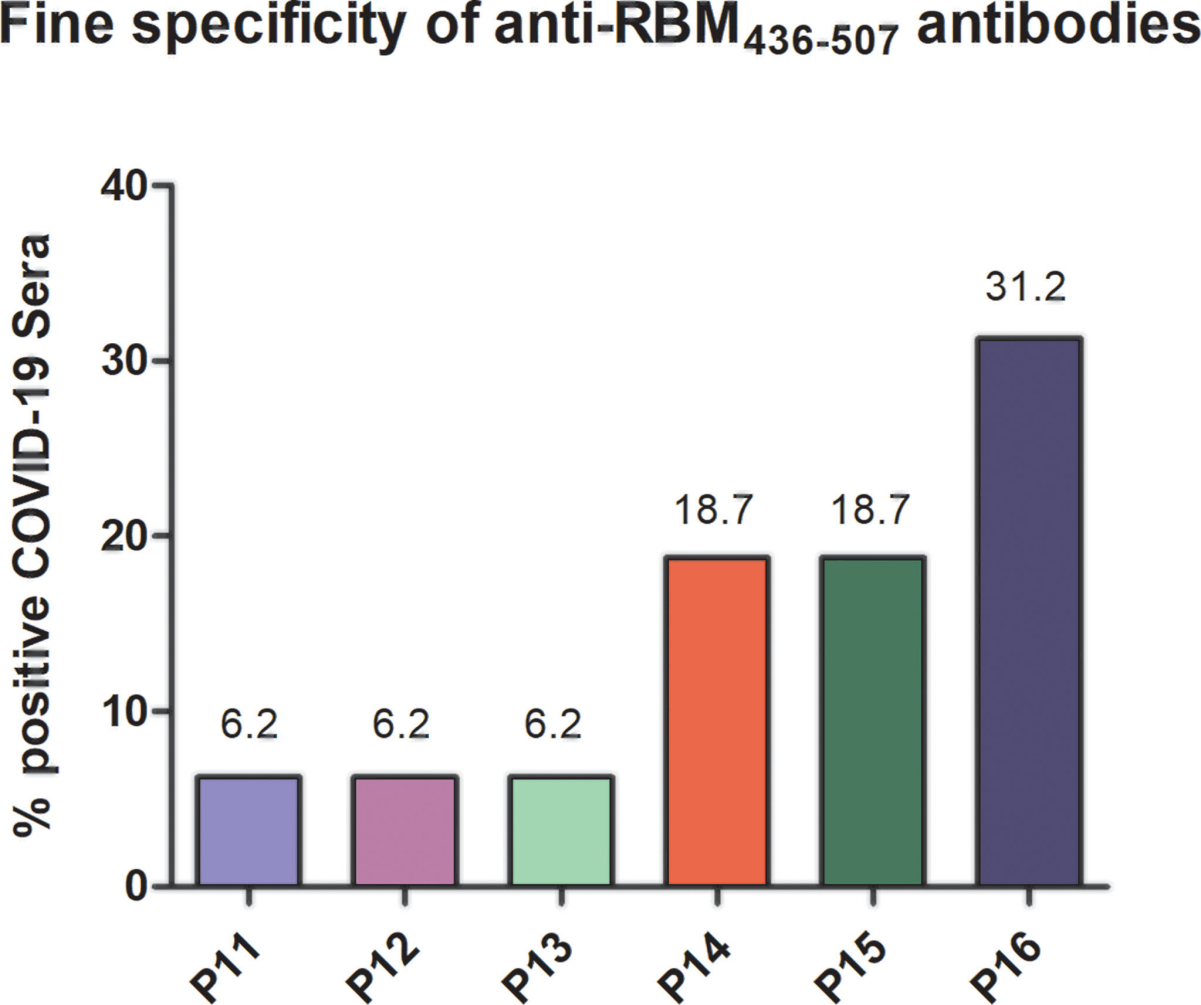
Fine specificity of anti-RBM436-507 antibodies in COVID-19 patients. Reactivity of anti-RBM positive COVID-19 sera with 20-mers overlapping peptides (P11-P16) covering the entire RBM_436-507_ sequence. Results are shown as percentage of anti-RBM positive sera reacting with the specific peptide.

**Figure 9:**
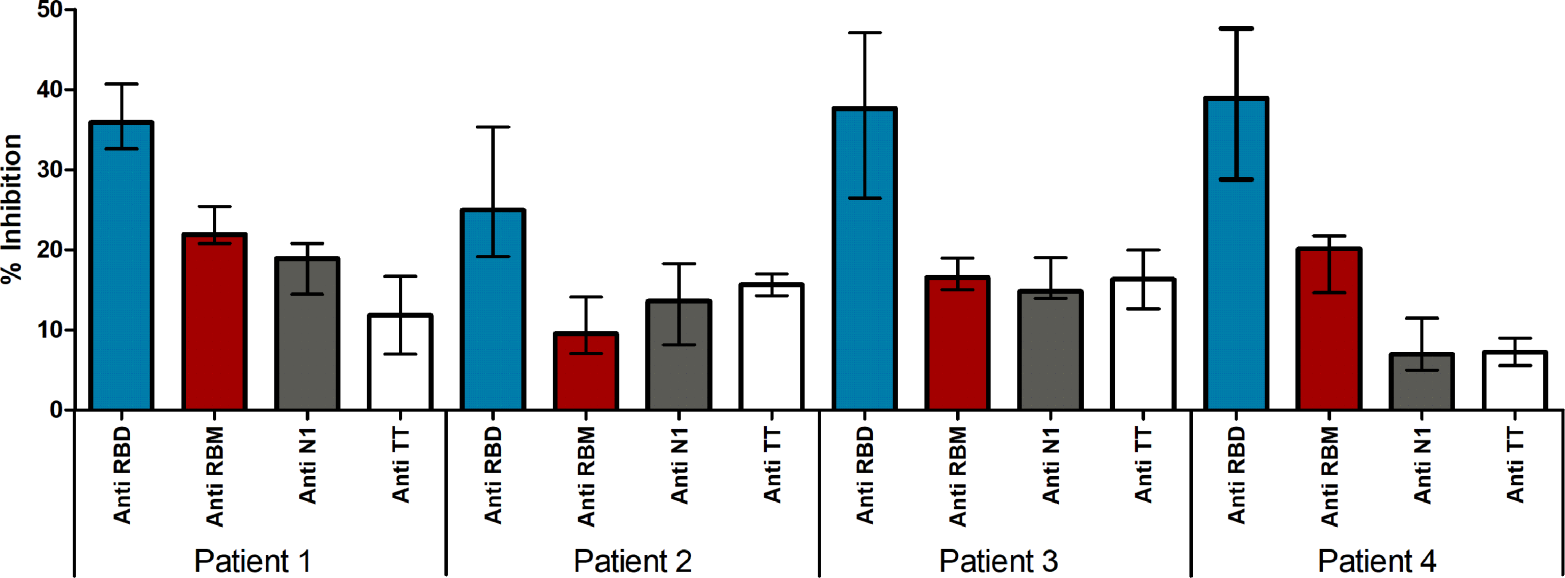
Neutralizing ability of antibodies in Covid-19 patients. Neutralizing ability of antigen eluted anti-S, anti-RBD and anti-RBM antibodies in COVID-19 patients. Results are shown as the percentage of inhibition of ACE-HRP binding to RBD.

Since neutralizing antibodies mostly specific for RBD but also to several targeted epitopes are produced during natural infection (21, 22), in the ACE2-RBD binding neutralization assay, antibodies to the whole RBD and to RBM_436-507_ were compared. In the case of humans with confirmed COVID-19 infection, sera positive to RBM_436-507_ were tested using the 20-mer overlapping peptides covering the entire RBM sequence (**Table 1**). As shown in **Figure 8**, immune response mainly targets the N terminal domain (P15-P16) rather than the C-terminal part (P11-P12). To evaluate the ability of antibodies to RBD or RBM_436-507_ sequences to block ACE2 binding to RBD, specific anti-RBD and anti-RBM_436-507_ antibodies were eluted from COVID-19 positive sera using antigen-coated wells and incubated with labeled ACE2 on solid-phase RBD. Anti-RBD antibodies eluted from 4 COVID-19 sera inhibited the binding of labeled ACE2 to solid-phase RBD (**Figure 9**). Anti-RBM_436-507_ antibodies from 2 out of 4 sera displayed some inhibition, higher than anti-N1 and anti-TT control antibodies.

## Discussion

This study confirmed the high seroreactivity of the full-length S and RBD recombinant proteins and described the immunogenicity of the synthetic RBM_436-507_ fragment. Moreover, it compared the antibody responses induced by natural human exposure to SARS-CoV-2 with that of rodents experimentally immunized with the three antigens.

Analysis of 3D structures of the S protein and RBD-ACE2 complex led to selecting a RBD 72 aa long segment (RBM_436-507_) highly specific to SARS-CoV-2 and located in the RBD-ACE2 interface. Importantly, *in silico* studies confirmed the presence in this protein fragment of multiple immune epitopes (B- and T-cell epitopes) previously identified (33), and our CD data suggested that the RBM_436-507_ peptide alone can partially preserve the extended beta-conformation observed in the context of the native protein structure. Indeed within the folded Spike protein, two short antiparallel beta sheets are observed (residues 453-454, 492-493 sheet1, and 473-474, 488-489, sheet2) but many other residues are in extended conformation (38/72, 53 %). The RBM_436-507_ peptide shows a high percentage of random coil conformation (about 50%) as expected for an isolated peptide; however, it maintains about one half of the extended conformation of the segment 436-507 when included in the whole protein which is an interesting result especially considering that many of the epitope residues of RBM that make interactions with a human neutralizing antibody (P2B-2F6 Fab) are in extended conformation, notably K444, N448, L452, V483, E484, F490 and S494 (38)

These features, together with the high RBD immunogenicity during human natural infection, vaccination and animal immunization, as well as the efficient neutralization of the RBD-ACE2 interaction by anti-RBD antibodies, encouraged the search for a smaller fragment with vaccine potential, suitable for production by peptide synthesis technology. It was hoped that the smaller fragment could elicit virus-neutralizing antibodies with similar or superior vaccine performance than the S protein.

The multiple vaccines delivered worldwide are based on the full-length S protein using different technological platforms (39). Although most of them have displayed high protective efficacy, their effectivity, particularly the antibody response's longevity and the virus-neutralizing function, appears short-lasting. Within less than a year of a two doses immunization schedule, a third vaccine dose was required to maintain the protection level; moreover, boosting vaccine doses may be further required to offer functional immunity in the population (40). Because of the vast virus propagation capacity in the population, frequent vaccination generates a significant logistic and global economic challenge; therefore, alternative vaccine platforms are envisioned.

The strong positive correlation of the ELISA seroreactivity of the S (45% = 29/64) and RBD (53% = 34/64) proteins (p<0,0001) is very interesting and confirms the feasibility of using a fragment of the S protein as vaccine. In addition, this result correlates with the highly efficient neutralization induced by mouse anti-S and -RBD sera. Moreover, the IgG ELISA reactivity of these two proteins with COVID19 and pre-pandemic normal sera (>97.5^th^ percentile) indirectly confirmed the response specificity to SARS-COV-2. In contrast, specific IgM and IgA antibodies are less frequent in COVID-19 patients. This latter finding may be explained because in the COVID-19 sera the primary IgM response and IgA had waned. These results support the idea that shorter protein fragments i.e., RBD would have the capacity to stimulate at least a similar immune response to S protein.

We show here that the antibody recognition of RBM_436-507_ as an isolated fragment, i.e., as RBM_436-507_ peptide, was present in a fraction of COVID-19 donors. In COVID-19 patients, a polyclonal anti-RBM_436-507_ antibody response with IgM, IgG and IgA isotypes was detected in one third of the cases, in amounts correlated with the level of anti-RBD and anti-S antibodies. Our finding that COVID-19 patients recognise RBM_436-507_ and smaller peptides within this sequence is in agreement with a report from 2020 (17) showing that infected subjects produced antibodies to multiple sequences, such as S_412-431_ and S_446-465_, that overlap ACE2 contact residues, and S_432-451_ and S_475-494_, that are adjacent to critical residues contacted by ACE2, all contained within RBM_436-507_.

The high level of neutralization achieved by mice sera after the first immunization dose with RBD encouraged selection of a smaller protein fragment with vaccine potential. Complete neutralization is produced after the first immunization with RBD, whereas similar neutralization by anti-S antibodies is only obtained after two immunization doses. In contrast, the poor neutralization of the anti-RBM_436-507_ antibodies was unexpected and deserves further studies. This result is surprising as there was significant cross reactivity of anti-RBD and anti-RBM_436-507_.

The neutralizing activity of anti-RBM_436-507_ antibodies might be associated with the lack of recognition of the full-length S by the anti-RBM_436-507_ sera. In addition, the high immunogenicity of RBM_436-507_ mice confirms the presence of T-cell epitopes within this protein segment, as suggested by the analysis performed by Grifoni et al. (33).

Mouse IgG antibodies efficiently reacted with both RBM_436-507_ and RBD, but not with S. The latter results can be explained by the fact that RBM_436-507_ represent only 6-7% of the whole protein. Moreover, anti-RBM_436-507_ specific antibodies elicited by mice immunization only partially inhibited (30-40%) the RBD-ACE2 interaction, while mouse anti S and RBD recognized RBM and induced 100% inhibition of the ligand-receptor interaction. These results suggest that the conformation of isolated RBM_436-507_ only partially overlaps with the RBM structures present in S or RDB. The relatively high percentage of β-strand conformation suggests that RBM_436-507_ peptide alone can partially preserve the extended conformation displayed along most of its sequence within the folded S protein (pdb code 6VXX) (21, 23).

In conclusion, our comparative analysis of immunological properties has shown that although RBM_436-507_ had reduced seroreactivity compared to the S protein and RBD, it could still represent an alternative path for developing virus control means, such as vaccines. The basis for this potential lies in its small size, absence of folding problems, possibility to constraint the RBM conformation in a required state, easy incorporation in different multimeric carriers and advantages associated with peptide synthesis production.

Further studies are needed to strengthen the potential use of RBM_436-507_ in vaccination strategies.

## Data Availability Statement

The raw data supporting the conclusions of this article will be made available by the authors, without undue reservation.

## Ethics Statement

The studies involving human participants were reviewed and approved by Comitato etico Area Vasta Nord Ovest (Pisa - Italy) Approval N° 17522. Comitè de Etica Centro Internacional de Vacunas Approval N° 04-2020. The patients/participants provided their written informed consent to participate in this study. The animal study was reviewed and approved by Comité de Etica Centro International de Vacunas Approval N° 04-2020.

## Author Contributions

Conceptualization: GC, AP, AK, and SH; Formal analysis: GC, MA-H, AP, AK, and SH; Investigation: GC, PR, FP, PM, LP, FE, AC, IP-M, SB, DK, MD, SQ, MA-H, and SH; Methodology: MA-H, FP, FE, SH, and GC; Project administration: MA-H and GC; Resources: GC, SH, AK, and AP; Supervision: MA-H, SH, and GC; Validation: MA-H, GC, and AK; Visualization: MA-H, SH, and GC; Writing: PM, PR, GC, SH, FP, and AK. All authors contributed to the article and approved the submitted version.

## Funding

This work was funded by MVDC/CIV Foundation (grant 150820) and by the Italian Ministry of Health grant COVID-2020-12371849

## Conflict of Interest

The authors declare that the research was conducted in the absence of any commercial or financial relationships that could be construed as a potential conflict of interest.

## Publisher’s Note

All claims expressed in this article are solely those of the authors and do not necessarily represent those of their affiliated organizations, or those of the publisher, the editors and the reviewers. Any product that may be evaluated in this article, or claim that may be made by its manufacturer, is not guaranteed or endorsed by the publisher.

## Supplementary Material

Supplementary Figure 1 ELISA titration of the mouse antibody response. Humoral response induced in mice after immunization at days 0, 20, and 40: ELISA used three-fold serial dilutions of sera, starting at 1:100. Antibody titers are expressed as O.D

Supplementary Figure 2 Effect of pH on anti-Spike and -RBD antibodies. Distribution of Anti-Spike antibodies **(A)** and anti-RBD antibodies **(B)** analyzed by ELISA under acid conditions (pH 5) and neutral conditions (pH 7.4). Results are expressed as OD 450 nm.
