# Expression of concern on: A SARS–CoV-2 spike receptor binding motif peptide induces anti-spike antibodies in mice andIs recognized by COVID-19 patients

**DOI:** 10.3389/fimmu.2023.1292353

**Published:** 2023-09-15

**Authors:** Frontiers Editorial Office

With this notice, Frontiers states its awareness of serious allegations surrounding the institutional review board Centro Internacional de Vacunas cited in this article. These allegations are being investigated in line with COPE guidelines. The situation will be updated as soon as the investigation is complete.

