# Supplementary figures and images for: A SARS–CoV-2 Spike Receptor Binding Motif Peptide Induces Anti-Spike Antibodies in Mice andIs Recognized by COVID-19 Patients
Source: Front Immunol. 2022 May 26;13:879946. doi: 10.3389/fimmu.2022.879946 (PMC9178084; doi:10.3389/fimmu.2022.879946)

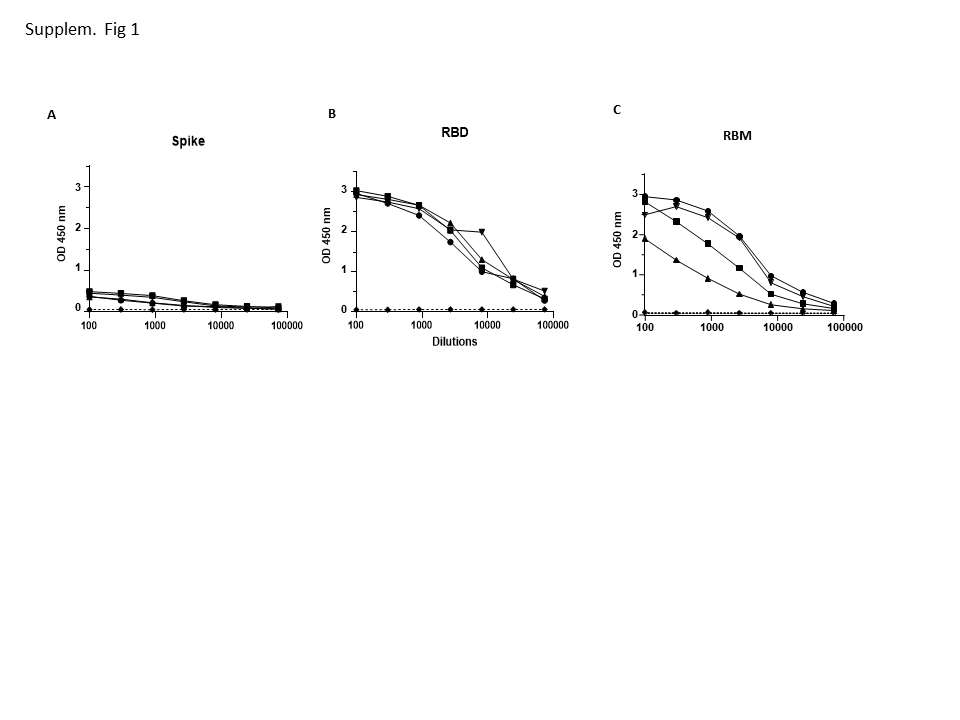

Supplement: Supplementary Figure 1 — ELISA titration of the mouse antibody response. Humoral response induced in mice after immunization at days 0, 20, and 40: ELISA used three-fold serial dilutions of sera, starting at 1:100. Antibody titers are expressed as O.D [file Image_1.tif]

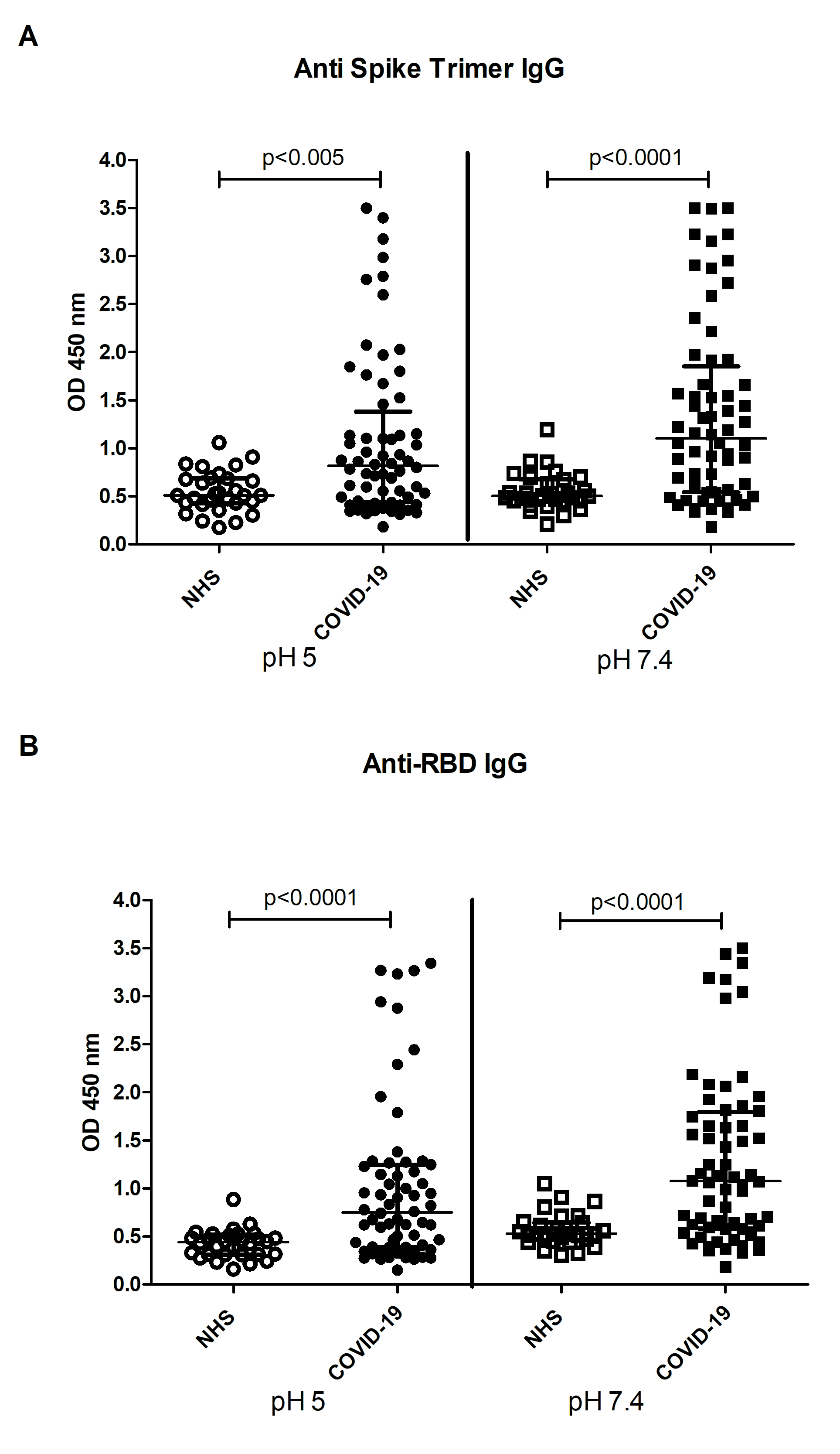

Supplement: Supplementary Figure 2 — Effect of pH on anti-Spike and -RBD antibodies. Distribution of Anti-Spike antibodies (A) and anti-RBD antibodies (B) analyzed by ELISA under acid conditions (pH 5) and neutral conditions (pH 7.4). Results are expressed as OD 450 nm. [file Image_2.tif]
