# Supplementary material for: A SARS–CoV-2 Spike Receptor Binding Motif Peptide Induces Anti-Spike Antibodies in Mice andIs Recognized by COVID-19 Patients
Source: Front Immunol. 2022 May 26;13:879946. doi: 10.3389/fimmu.2022.879946 (PMC9178084; doi:10.3389/fimmu.2022.879946)
Supplement: Supplementary file 3 [file DataSheet_1.pdf]

A SARS–CoV-2 Spike Receptor Binding Motif peptide induces anti-spike antibodies in mice and is recognized by COVID-19 patients

SUPPORTING INFORMATION

Peptide synthesis, purification, and characterization.

Materials. Peptide grade *N,N*-dimethylformamide (DMF), all Fmoc protected amino acids were purchased from Sigma Aldrich (Milan, Italy). TentaGel® R RAM resin was purchased from Rapp Polymere GmbH (Tuebingen, Germany). Activators *N,N*′-diisopropylcarbodiimide (DIC), and oxima pure were purchased from Sigma Aldrich (Milan, Italy). Trifluoroacetic acid (TFA), triisopropyl silane (TIS), and 2,2′-(ethylenedioxy)diethanethiol (DODT), diisopropyl ether (iPr_2O), 2-propanol, ammonium hydroxide 30% (v/v) in water solution, hydrogen peroxide 30% (v/v) in water solution and HPLC plus water were purchased from Sigma Aldrich (Milan, Italy). HPLC-grade acetonitrile (CH_3CN) was purchased from Carlo Erba (Milan, Italy).

Peptide synthesis. Peptides were prepared by microwave-assisted solid-phase peptide synthesis (MW-SPPS) and were assembled using an optimized protocol based on the Fmoc/tBu strategy and N,N′-diisopropylcarbodiimide (DIC) / Oxyma Pure as coupling reagents on the automated MW-assisted synthesizer Liberty Blue™ (CEM, USA). We used a Tentagel R RAM resin, specially designed for long and difficult peptide sequences, based on a PEG polymer that space out growing peptide sequences, thus reducing steric problems, and featuring a low loading (0.18 mmol/g). For this reason, we used a high swelling protocol, specifically optimized in our laboratory.

The Fmoc/tBu MW-SPPS cycle consisted of: 1) swelling in DMF for 30 min; 2) Fmoc-deprotection by 20% (v/v) piperidine/DMF (4 mL, 81 equiv); 3) washings with DMF (4 × 4 mL); 4) double coupling with the Fmoc-protected amino acids (5 equiv, 0.2 M in DMF), Oxyma Pure (5 equiv, 1 M in DMF), and DIC (5 equiv, 0.5 M in DMF); 5), washings with DMF (2 × 4 mL). Peptide elongation was performed by repeating the MW cycle for each amino acid coupling and deprotection. In the case of the 72-mer $RBM_{436-507}$, starting from cycle 43 the molar excess of the couplings was switched from 5 to 10 equiv and the swelling volume was also doubled. Both deprotection and coupling reactions were performed at 90 °C in a Teflon vessel, applying microwave energy under nitrogen bubbling and monitoring the reaction temperature by an internal fiber-optic sensor. After the coupling of the last residue, the resin was filtered, washed with DMF (3 × 4 mL) and 2-propanol (3 × 4 mL), and dried under vacuum to obtain the dry peptide-resin.

Cleavage from the resin. The cleavage of the crude peptides from the resin, with concomitant deprotection of acid sensitive amino acid side-chains, was achieved by treatment of the peptide-resin with the cocktail TFA/DODT/H_2O/TIS (10 mL, 94:2.5:2.5:1) for 4.5 h at room temperature under soft stirring. The resin was filtered and rinsed with fresh TFA. The peptide was precipitated from the cleavage mixture by addition of ice-cold iPr_2O (40 mL). The solid was isolated by centrifugation and dried under vacuum. Characterization of the crude thiol free linear precursor peptides was performed by analytical UHPLC-ESI-MS.

Disulfide bond formation ($RBM_{436\text{-}507}$ and P12). The crude peptides were introduced in a round bottom flask and a mixture of water and CH_3CN (1:1) was added, stirring for about 15 minutes. After complete dissolution, additional water was added to the reaction mixture to obtain a final concentration of 3.3 mg/mL. pH was adjusted to 8.5-9.5 adding NH_4OH 7.5 %. 0.1 mL per gram of crude peptide and then Hydrogen Peroxide (30% v/v water solution) was added to the solution. After 1 h of magnetic stirring at room temperature, the reaction was quenched adding Formic Acid to adjust the pH to 3 and the reaction mixture was lyophilized without further evaporation.

Purification and analysis. After cleavage (P11, P13-P16) or after oxidation ($RBM_{436\text{-}507}$ and P12), purifications were performed by flash chromatography on a CombiFlash® NextGen 300+ Teledyne ISCO instrument with a Teledyne ISCO RediSep® Gold 15g column followed by semi-preparative chromatography in a HPLC Waters 600 coupled with Waters UV DAD 2487 with a Phenomenex Jupiter C4 column ($RBM_{436\text{-}507}$) or a Sepax Bio-C18 column to achieve a UHPLC purity grade >70%. ($RBM_{436\text{-}507}$ and P16) or >87% (P11-P15).

Final products were analytically characterized by liquid chromatography coupled with ESI single quadrupole mass spectrometry in a UHPLC-MS system using Acquity UPLC CSH™ C18 or C8 columns and/or by MALDI-ToF analysis (table S1).

Table S1. Peptides analytical data.

Name	HPLC Purity (%)	Molecular Weight (g/mol)
$RBM_{436-507}$	>70	8260.90
P11	94.7	2552.75
P12	95.8	2222.43
P13	87.6	2122.21
P14	93.1	2483.78
P15	93.0	2520.84
P16	>70	2431.62
